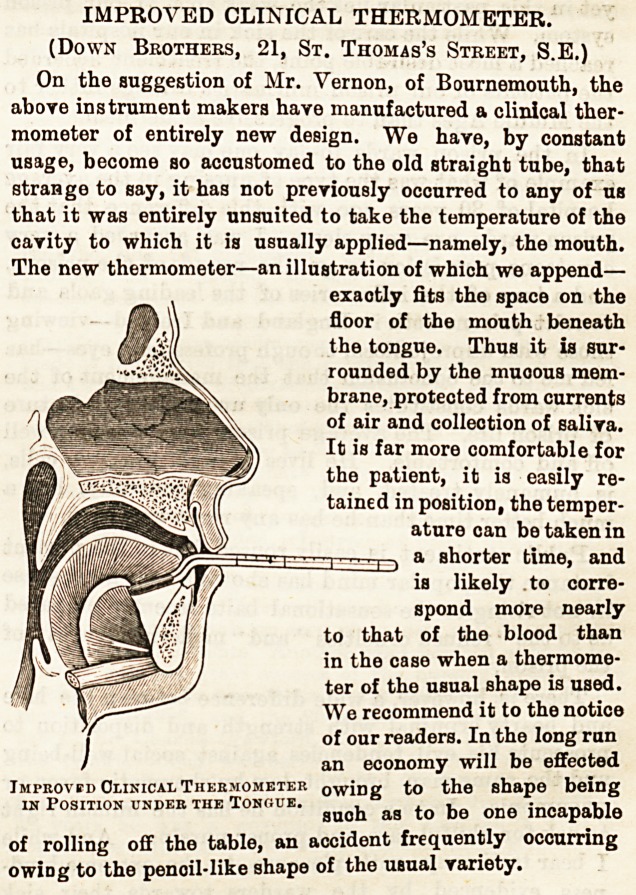# New Appliances and Things Medical

**Published:** 1898-06-04

**Authors:** 


					NEW APPLIANCES AND THINGS MEDICAL.
fWe shall be glad to receive, at our Office, 28 & 29, Southampton Street, Strand, London, W.O., from the manufacturers, specimens of all new
preparations and appliances which may be brought out from time to time.]
NEW INSPECTION BOWL.
(Down Brothers, 21, St. Thomas's Street, S.E.)
The glass bowl of which we
give an illustration has been
designed by Nurse Case, of
the Wolverhampton General
Hospital, for keeping and in-
specting the various Beoretions
or discharges from the body
which it may be the duty of
the physician or surgeon to
examine during the treatment
of a case. The contents can
be seen readily without remov-
ing the cover, the quantity can
be measured when necessary,
and the air-tight lid is of
special value when the sub-
stances are of an offensive or
dangerous character.
HYGIENIC PINE FIBRE MATTRESS.
(Atkinson and Co., 198, Westminster Bridge Road, S.E.)
This mattress is made of a fibre which is said to be derived
from the " needles " of the Long Leaf Pine. From these a
valuable pine oil is produced, and it would appear that after
the decoction a material is left from which this fibre is
obtained. It certainly is a clean and clean-smelling substance
for use in the making of mattresses, and, without being hard,
is very elastic. We have subjected it to considerable
pressure, which was maintained for many hours, but tho
fibre quickly sprang up again, nearly to its old position, when
the pressure was removed. It is not entirely free from dust,
but this dust doss not consist of an admixture of any foreign
material; in other words, it is not dirt, but only broken
fibre. An examination of the material shows that although
each fibre is clean and smooth?in fact, as clean and smooth
?as straw?the fibres as a mass possess considerable binding
power?they interlace and twist over one another in such a
way as to prevent their slipping away from the part of the
bedding on which pressure is applied. The only possible
objection to it is its inflammability. From their elasticity,
their cleanliness, and the ease with which the material of
which they are constructed can be cleaned by washing in hot
water, we think these mattresses admirably suited, not only
for hospitals, but for other institutions, such as hotels,
where the same beds have to be used by many people in
succession, and where it is desirable not to use any material
which is liable to harbour inseot life.
IMPROVED CLINICAL THERMOMETER.
(Down Brothers, 21, St. Thomas's Street, S.E.)
On the suggestion of Mr. Vernon, of Bournemouth, the
above instrument makers have manufactured a clinical ther-
mometer of entirely new design. We have, by constant
usage, become so accustomed to the old straight tube, that
strange to say, it has not previously occurred to any of us
that it was entirely unsuited to take the temperature of the
cavity to which it is usually applied?namely, the mouth.
The new thermometer?an illustration of which we append?
exactly fits the space on the
floor of the mouth beneath
the tongue. Thus it is sur-
rounded by the muoous mem-
brane, protected from currents
of air and collection of saliva.
It is far more comfortable for
the patient, it is easily re-
tained in position, the temper-
ature can be taken in
a shorter time, and
is likely to corre-
spond more nearly
to that of the blood than
in the case when a thermome-
ter of the usual shape is used.
We recommend it to the notice
of our readers. In the long run
an economy will be effected
owing to the shape being
such as to be one incapable
of rolling off the table, an accident frequently occurring
owiDg to the penoil-like shape of the usual Yariety.
QQWM Bnns lqndctn
IMPROVED CLINICAL THERMOMETER.
(Down Brothers, 21, St. Thomas's Street, S.E.)
On the suggestion of Mr. Vernon, of Bournemouth, the
above instrument makers have manufactured a clinical ther-
mometer of entirely new design. We have, by constant
usage, become so accustomed to the old straight tube, that
strange to say, it has not previously occurred to any of us
that it was entirely unsuited to take the temperature of the
cavity to which it is usually applied?namely, the mouth.
The new thermometer?an illustration of which we append?
exactly fits the space on the
floor of the mouth beneath
the tongue. Thus it is sur-
rounded by the muoous mem-
brane, protected from currents
of air and collection of saliva.
It is far more comfortable for
the patient, it is easily re-
tained in position, the temper-
ature can be taken in
a shorter time, and
is likely to corre-
spond more nearly
to that of the blood than
in the case when a thermome-
ter of the usual shape is used.
We recommend it to the notice
of our readers. In the long ran
an economy will be effected
Improved Clinical Thermometer owiDg to the shape being
IN POSITION UNDEB THE TONGUE. ^ ^ ^ ^ ^ incapable
of rolling off the table, an accident frequently occurring
owiDg to the penoil-like shape of the usual variety.

				

## Figures and Tables

**Figure f1:**
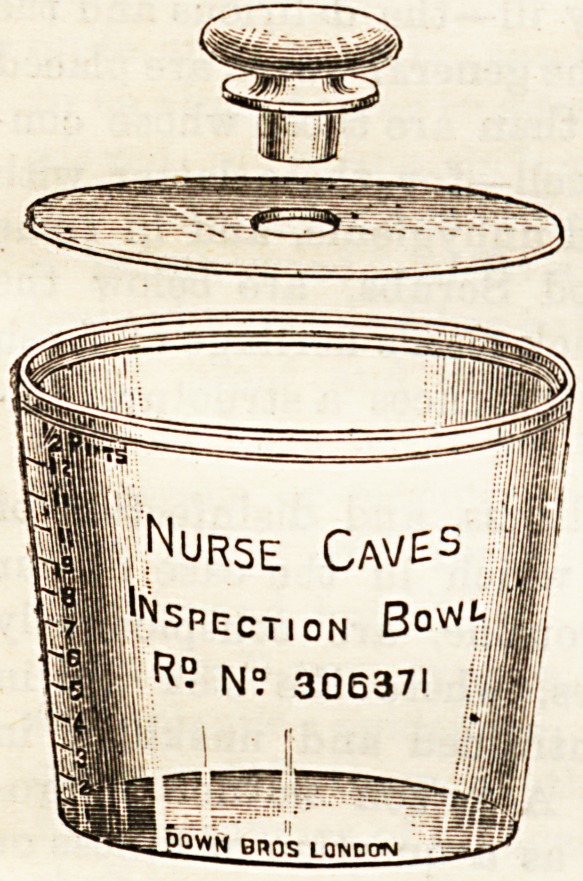


**Figure f2:**